# Chlorhexidine-Induced Contact Anaphylaxis

**DOI:** 10.7759/cureus.88598

**Published:** 2025-07-23

**Authors:** Federico Sandoval Morales, Fabien Pelletier, François Aubin, Florence Castelain

**Affiliations:** 1 Dermatology Department, Allergy Unit, Besançon University Hospital, Besançon, FRA

**Keywords:** allergy skin testing, allergy symptoms, chlorhexidine, chlorhexidine allergy, contact hypersensitivity, perioperative anaphylaxis

## Abstract

Perioperative anaphylaxis is a rare but life-threatening event, with chlorhexidine increasingly recognized as a significant trigger. Though commonly used as an antiseptic due to its broad-spectrum antimicrobial properties, chlorhexidine can lead to sensitization through repeated exposure, resulting in both immediate and delayed hypersensitivity reactions.

We report a case of severe perioperative anaphylaxis in a 72-year-old woman following abdominal surgery at the University Hospital of Besançon in May 2024. Chlorhexidine was used for preoperative skin antisepsis. Shortly after anesthesia induction with etomidate and rocuronium, the patient developed bronchospasm, followed by refractory hypotension and oxygen desaturation after administration of additional anesthetics. She required intensive care admission. Elevated acute serum tryptase (123 µg/L) confirmed anaphylaxis. Allergy investigations revealed positive skin tests and specific immunoglobulin E (IgE) to chlorhexidine, while tests for other agents were negative. Genetic testing excluded mast cell disorders or hereditary alpha-tryptasemia. Surgery was successfully completed five days later with chlorhexidine avoidance and no further reactions.

This case underscores the potential severity of chlorhexidine-induced perioperative anaphylaxis. Given the widespread use of chlorhexidine and the possibility of delayed or underrecognized sensitization, clinicians must remain vigilant, particularly in patients with prior unexplained reactions. Preoperative screening and heightened awareness are essential to reduce morbidity and mortality associated with this increasingly reported allergen.

## Introduction

Perioperative anaphylaxis, although rare, is a potentially life-threatening event, occurring in three to five cases per million procedures. The mortality rate ranges from 1.4% to 4.8%, which is higher than other causes of anaphylaxis [1]. The causes of severe anaphylaxis can vary based on geographical location and the timing of symptom onset [2,3].

Chlorhexidine, widely used as an antiseptic in medical settings due to its broad-spectrum activity, can cause sensitization through prolonged or repeated exposure, both in healthcare settings and via personal care products. Chlorhexidine allergy can manifest with varying severity, from mild skin reactions to severe anaphylaxis or contact dermatitis [4]. Both immediate and delayed hypersensitivity reactions can occur simultaneously in the same patient [5].

## Case presentation

We report a case of severe perioperative anaphylaxis triggered by chlorhexidine in a 72-year-old woman who underwent abdominal surgery at the University Hospital of Besançon in May 2024. Her medical history included unexplored hypersensitivity reactions to amoxicillin/clavulanic acid and Fucidin, hypertension, obesity, non-embolic atrial fibrillation, resolved hepatitis B, chronic venous insufficiency, hepatic steatosis, peripheral arterial disease, non-diabetic peripheral neuropathy, and a recent radius and ulna fracture. Additional comorbidities included a past smoking habit (now ceased) and chronic alcohol use. Her surgical history comprised varicose vein surgery and splenectomy.

Chlorhexidine was used for skin preparation, and anesthesia was induced with etomidate and rocuronium. Shortly after induction, bronchospasm occurred and was treated with inhaled salbutamol. Ten minutes later, the patient received remifentanil, ketamine, and propofol. However, 30 minutes after induction, she developed refractory hypotension, persistent bronchospasm, and oxygen desaturation, notably without any cutaneous involvement. The patient's condition improved after fluid and intravenous adrenaline administration. She was then transferred to the intensive care unit (ICU). Acute serum tryptase was elevated (123 µg/L vs. 9.33 µg/L at baseline), consistent with an anaphylactic reaction. Five days later, she underwent successful abdominal surgery without further reactions.

Allergy testing at the Allergy Unit of the University Hospital of Besançon included skin prick tests (SPT) and intradermal tests (IDT). The chlorhexidine SPT was initially negative, after which the IDT was performed. While the IDT was ongoing, the SPT became positive. Ultimately, both the chlorhexidine SPT and IDT were positive. Tests for other anesthetic medications and antibiotics were negative (Table 1). Chlorhexidine-specific immunoglobulin E (IgE) tests were positive at 0.75 KUA/L. Given the increased baseline tryptase levels, genetic testing was performed, which ruled out a mast cell disorder or hereditary alpha-tryptasemia.

**Table 1:** Results of the skin prick test (SPT) and intradermal test (IDT).

Compound	Concentration	SPT dilution	IDT dilution	SPT result	IDT result
Positive control	-	-	-	Positive	-
Negative control	-	-	-	Negative	Negative
Chlorhexidine	0.5%	5 mg/mL	10^-3^	Positive	Positive
Betadine	Pure	Pure	-	Negative	Negative
Latex	-	-	-	Negative	Negative
Sedative-hypnotic					
Propofol	10 mg/mL	-	10^-2^ to 10^-1^	Negative	Negative
Midazolam	5 mg/mL	-	10^-2^ to 10^-1^	Negative	Negative
Etomidate	2 mg/mL	-	10^-3^ to 10^-2^	Negative	Negative
Ketamine	10 mg/mL	-	10^-3^ to 10^-1^	Negative	Negative
Lidocaine	10 mg/mL	-	10^-3^ to pure	Negative	Negative
Opioids					
Morphine	10 mg/mL	10^-1^	10^-4^	Negative	Negative
Fentanyl	0.05 mg/mL	-	10^-3^ to 10^-2^	Negative	Negative
Remifentanil	1 mg/mL	-	10^-2^ to 10^-1^	Negative	Negative
Sufentanil	50 µg/mL	-	10^-2^ to 10^-1^	Negative	Negative
Curares					
Atracurium	10 mg/mL	10^-1^	10^-4^ to 10^-3^	Negative	Negative
Cisatracurium	10 mg/mL	-	10^-4^ to 10^-2^	Negative	Negative
Mivacurium	2 mg/mL	10^-1^	10^-4^ to 10^-3^	Negative	Negative
Rocuronium	10 mg/mL	-	10^-2^	Negative	Negative
Suxamethonium	10 mg/mL	-	10^-2^	Negative	Negative
Antibiotics					
Amoxicillin/clavulanic acid	200/25 mg/mL	-	10^-3^ to 10^-1^	Negative	Negative
Fusidic acid	250 mg	-	-	Negative	-
Metronidazole	5 mg/mL	-	10^-3^ to 10^-1^	Negative	Negative
Gentamicin	10 mg/mL	-	10^-3^ to 10^-1^	Negative	Negative

## Discussion

This case highlights the diagnostic challenges associated with perioperative anaphylaxis, which often requires multiple drug skin tests and specific IgE assays when available. In our case, despite an initially negative chlorhexidine skin prick test, a delayed positivity was observed after 35 minutes of exposure. The combined use of skin prick tests, intradermal tests, and chlorhexidine-specific IgE assays, each contributing to increased diagnostic sensitivity [6], was essential in confirming the diagnosis, highlighting that reliance on a single test may result in missed cases.

It also underscores the potential severity of chlorhexidine allergy. Although the sensitization rate appears low, perioperative anaphylaxis due to chlorhexidine remains among the top five causes globally, with some variation depending on geographic regions [7]. Topical exposure to chlorhexidine, while less common than intravenous exposure, accounts for up to 16% of perioperative anaphylaxis cases [7]. Contact hypersensitivity is often overlooked and likely underreported as a cause of anaphylaxis in clinical practice.

Moreover, the elevated baseline serum tryptase level prompted a comprehensive evaluation for potential underlying mast cell disorders. Genetic testing excluded hereditary alpha-tryptasemia [8] as well as the presence of the D816V mutation in the c-kit gene, which is frequently associated with systemic mastocytosis [9]. This underscores the importance of thorough patient assessment when elevated baseline tryptase is detected in the context of anaphylaxis.

Given the widespread and increasing use of chlorhexidine in both healthcare and personal care products, a growing population is at risk for sensitization and severe allergic reactions. Clinicians must maintain a high index of suspicion for chlorhexidine allergy, especially since symptoms may develop with delayed onset compared to intravenously administered agents. Preoperative screening for prior allergic reactions and chlorhexidine exposure should be incorporated into routine patient assessments to reduce the risk of perioperative anaphylaxis [6].

## Conclusions

This case illustrates several critical points in the diagnosis and management of perioperative anaphylaxis related to chlorhexidine allergy. First, it emphasizes that initial negative skin prick tests do not exclude chlorhexidine sensitization, as delayed positivity can occur, necessitating prolonged monitoring during allergy testing to avoid false-negative results. Second, the finding of elevated baseline serum tryptase levels should not be overlooked; it warrants comprehensive evaluation to exclude underlying mast cell disorders such as hereditary alpha-tryptasemia or systemic mastocytosis, which may influence patient risk and management strategies. Third, the increasing use of chlorhexidine in healthcare and personal care settings raises the likelihood of sensitization in the general population, underscoring the need for heightened clinical awareness. Preoperative screening protocols should routinely include detailed histories of allergic reactions and potential chlorhexidine exposure, coupled with appropriate diagnostic testing when indicated. Ultimately, these measures are essential to improve patient safety by preventing severe and potentially life-threatening perioperative allergic reactions.
